# Biodiversity and Microbial Resistance of Lactobacilli Isolated From the Traditional Greek Cheese Kopanisti

**DOI:** 10.3389/fmicb.2018.00517

**Published:** 2018-03-22

**Authors:** Georgios Rozos, Chrysa Voidarou, Elisavet Stavropoulou, Ioannis Skoufos, Athina Tzora, Athanasios Alexopoulos, Eugenia Bezirtzoglou

**Affiliations:** ^1^Laboratory of Microbiology, Biotechnology and Hygiene, Faculty of Agricultural Development, Democritus University of Thrace, Orestiada, Greece; ^2^Faculty of Agricultural Technology, Food Technology and Nutrition, Department of Agricultural Technology, Technological Educational Institute of Epirus, Arta, Greece; ^3^Service des Urgences, Centre Hospitalier Universitaire Vaudois, Lausanne, Switzerland; ^4^Medical School, Democritus University of Thrace, Alexandroupolis, Greece

**Keywords:** biodiversity, microbial, resistance, lactobacilli, Greek, cheese, Kopanisti

## Abstract

Kopanisti is a Greek artisan cheese produced from raw milk in the island of Mykonos, Greece. The milk is left to rest for 12–24 h and then the rennet is added. After its formation the curd is left to drain for 2–3 days and is ready either for consumption (as tyrovolia fresh cheese), or with the addition of extra salt, the curd is left to ripen through further fermentation and surface development of Penicillium fungi, aprocess leading to the production of the traditional Greek cheese Kopanisti. From 120 samples of kopanisti, 574 *Lactobacillus* strains were isolated, distributed in 17 species (16 of them isolated from tyrovolia as well). Strains from 15 species were found resistant or multiresistant against 15 antimicrobial agents, representing all categories of antibiotics. Analysis revealed that the resistance was moderated during ripening of the curd from tyrovolia to Kopanisti. Resistance against penicillin G, ampicillin/sulbactam, clindamycin, chloramphenicol, streptomycin, trimethoprim, metronidazole, vancomycin, teichoplanin, and quinupristin/dalvopristin was significantly enhanced, while the resistance against ampicillin, erythromycin, oxytetracycline, gentamycin, and fucidic acid was significantly reduced. These changes during ripening suggest that resistance to antimicrobials is a dynamic process subjected to environmental factors. The biodiversity of isolated *Lactobacillus* strains is impressive and explains the exquisite sensorial characteristics of the cheese. However, the extent of the resistance is alarming.

## Introduction

Kopanisti is a traditional artisan cheese produced in the Cyclades island complex in the central Aegean archipelago in Greece. The cheese is made from raw milk originating from ewes and goats and in some cases from cows. The milk is left for a few hours and then rennet and salt are added. After its formation the curd is left to ripe for 2–3 weeks (personal interview with the producers). During ripening more salt is added and the curd is thoroughly mixed on weekly basis. The major populations of the cheese's flora consist of lactobacilli and *Penicillium* spp. The cheese has a sharp intense characteristic flavor and is considered as local delicacy and has many culinary uses. It is part of the cultural identity of the area and of course tourists are encouraged to taste it.

Mykonos is a small island but is a worldwide known touristic destination. Hundreds of thousands of visitors swarm the island every year. Since Kopanisti is an artisan cheese made in households dispersed all over the island, the microbial resistance to antibiotics of the natural microflora of the cheese could reflect the impact of the touristic development on the microbial ecosystems of the island. The same result is expected by the use and misuse of antibiotics used in the clinical veterinary practice.

Lactobacilli are present in the alimentary tract of humans and animals as well as in various niches in nature and of course in all fermented foods such as the dairy products. Lactobacilli play an important role in the physiology of the gut and their action is recognized as to promote good health. However, the proximity of many species in the alimentary tract of the consumer is considered as a favorable precondition for the transfer of resistance to antibiotics from lactobacilli to other commensals or pathogens bacteria (Karapetkov et al., 2011). This transfer is horizontal and is achieved with the aid of plasmids and transposons (Dec et al., 2017).

Thus, it becomes of significant importance to assess the potential foodborne resistance, given that the local population and the visitors consume the cheese. It is of growing concern the possibility that genes controlling resistance to antibiotics could disseminate from food to the consumers' intestinal flora and be transferred horizontally to a pathogen (Lord Soulsby of Swaffham Prior, 2008; EFSA, 2011; CDC Antimicrobial Resistance, 2017). This is the first study of its kind that investigates the resistance to antibiotics of the lactobacilli of the microflora of Kopanisti to such an extent (number of samples and number of antibiotics). Furthermore, the results of this study test the hypothesis that artisan dairy products are sensitive indicators of the microbial resistance of their environment and this resistance can be studied in the microflora of the cheese.

## Materials and methods

A total of 120 samples from traditional artisan Greek cheese Kopanisti were collected from local producers, directly from their facilities. Samples were collected under normal conditions and immediately packaged in sterile polythene bags that were sealed and transported to the laboratory in cooler boxes containing ice packs where they were immediately stored in a cold chamber at a temperature of 2–4°C until analysis.

For isolation of bacteria, the rind of each cheese Kopanisti was removed and 10 g of the remainder cheese sample was homogenized in 40°C warm peptone water (10 g L^−1^ peptone from casein, 5 g L^−1^ sodium chloride, 20 g L^−1^ tri-sodium citrate dehydrate, pH 7.0) for 3 min in a stomacher blender. Then serial dilutions of the above homogenate were plated (a) on modified decarboxylating agar containing histidine (MDA-H) and (b) on 10 mL of MRS (de Man, Rogosa and Sharpe) broth (Difco Laboratories, Detroit, Mich., USA). After 24 h of anaerobic growth (37°C), 0.02 ml of the diluted solutions was spread for 48 h on MRS agar media. The isolated LAB were subjected for gram-staining, catalase, oxidase and motility tests; homo-fermentative and hetero-fermentative tests were performed in TSI agar slants as well as in sugar fermentation broth, with Durham's tube but replacing sorbitol by dextrose discs (Mandal et al., 2012). The non-motile gram-positive non-spore forming rods showing negative test results for catalase and oxidase production were considered as *Lactobacillus*. The lactobacilli thus obtained were grown in MRS broth at 42°C, and tested further for their identification up to the species level performing cultural, biochemical and sugar fermentation tests following Bergey's Manual of Determinative Bacteriology. A total of 574 *Lactobacillus* strains were isolated, distributed in 17 species. For long-term maintenance, the isolates were stored at −65°C in De Man, Rogose and Sharpe (MRS) supplemented with 20% (v/v) glycerol. Before analysis, the strains were routinely cultured at 37°C on MRS (Merck, Darmstadt, Germany) from which fresh cultures were prepared for inoculation of the final broth used for antimicrobial susceptibility testing.

### Determination of minimal inhibitory concentration

All antimicrobial agent powders were obtained from Sigma-Aldrich (Poland). Inocula were prepared by suspending fresh cultures in 0.9% NaClM so that the optical density (OD) of the suspension at 600 nm was 0.5. For *Lactobacillus* isolates, VetMIC 96-well microtiter plates (National Veterinary Institute, Uppsala, Sweden) were used for determining the MICs of the antimicrobial agents: Penicillin G (0.03 – 64 μg ml^−1^), Ampicillin (0.03 – 64 μg ml^−1^), Sulbactam/ampicillin (0.03 – 32 μg ml^−1^), Teicoplanin, Clindamycin (0.03 – 8 μg ml^−1^), Erythromycin (0.03 – 64 μg ml^−1^), Gentamicin (0.03 – 500 μg ml^−1^), Streptomycin (0.03 – 500 μg ml^−1^), Chloramphenicol (0.03 – 256 μg ml^−1^), Oxytetracycline (0.03 – 256 μg ml^−1^), Fucidic acid (0.03 – 256 μg ml^−1^), Teichoplanin (0.03 – 500 μg ml^−1^), Metronidazole (0.25 – 500 μg ml^−1^), Trimethoprim (0.03 – 500 μg ml^−1^), Quinupristin/dalfopristin(0.03 – 16 μg ml^−1^), and Vancomycin (0.03 – 256 μg ml^−1^). Microdilution plates were inoculated with 50 μl of a 1:500-diluted (in LSM broth) inoculum and 50 μl of the appropriate antibiotic concentration (stock solution previously dissolved in LSM), resulting in the final range of concentrations as described above. The antimicrobial agents used are indicated in Table S3. After this step the plates were incubated under anaerobic conditions at 37°C for 48 h. Subsequently, MICs were read as the lowest concentration of an antimicrobial agent at which visible growth was inhibited As positive control, a *Enterococcus faecalis* (ATCC 29212) strain was used (Klare et al., 2005).

The breakpoints for testing lactobacilli are not reported in the European Committee on Antimicrobial Susceptibility Testing (EUCAST) guidelines, and the procedure proposed by the CLSI (M-45) are not adapted to the growth requirements of all lactobacilli. In the present study the subpopulations of the strains of a species that grew in not consecutive MICs were considered as resistant and the MIC. As breakpoints were considered the MICs after which the resistant subpopulations grew.

The Spearman test (in a significance level of *p* < 0.05) was used to asses various correlations.

## Results

Table S1 shows the resistant strains while Table S4 shows the breakpoints in which the resistance was recorded. Table S3 shows the distribution of the resistant strains with respect to the various groups of antibiotics based on the mechanisms of action.

In total 574 strains were isolated, belonging to 17 species. Except *L. reuteri* and *L. casei* subspecies *pseudoplantarum*, the other 15 species included strains resistant to at least one antimicrobial compound. Resistance was observed against all the antibiotics (Klare et al., 2007) under study.

The distribution of resistance and multiresistance varied among the different *Lactobacillus* species as shown in Table S2. Resistance and multiresistance were expressed in various phenotypes even within the strains of the same species. The number of isolated strains of a species was correlated to the number of antibiotics against which resistance was observed (*r* = 0.634, *p* < 0.05), to the number of resistant and multiresistant strains (*r* = 0.953, *p* < 0.001 and *r* = 0.914, *p* < 0.001 respectively) and to the number of the different expressed phenotypes (*r* = 0.866, *p* < 0.001). It is rather alarming the fact that the majority of the isolated lactobacilli were found multiresistant (366 strains, 63.76%). Multiresistance—as isolated multiresistant strains within a species—was also correlated to the number of the expressed phenotypes (*r* = 0.902, *p* < 0.001), to the number of resistant strains (*r* = 0.910, *p* < 0.001) and to the number of antibiotics against which resistance was observed (*r* = 0.788, *p* < 0.001).

## Discussion

The resistance of *Lactobacillus* against various antibiotics has been investigated by many researchers in the past years. This genus is virtually omnipresent in nature, including humans, animals, plants, sewage, and wherever there are available carbohydrates for fermentation (Felis and Dellaglio, 2007; Montville and Matthews, 2008). Their importance to the dairy industry has been well-documented, while lactobacilli also play an important role as probiotics and are incorporated in various functional foods (Chamba and Irlinger, 2004; Bernardeau et al., 2006; Ugarte et al., 2006). Recently the interest of the microbial resistance of lactobacilli has been associated with the possibility of transferring of this resistance to the consumers of dairy and other products which contain resistant lactobacilli (Maragkoudakis et al., 2006).

In our study *Lactobacillus* originated from the Kopanisti cheese, produced in the island of Mykonos, Greece. The biodiversity of lactobacilli was impressive given that 17 species were involved and can be attributed to the different ecosystems established in the different households. Specific characteristic of every household (e.g., the number of animals, the animal species bred, the microclimate, etc.,) work as filters which permit to certain microorganisms to thrive while preventing others. Thus, the ecological niches in a household may act as amplifiers of the resistance to antibiotics if the thriving bacteria carry resistance genes (Mayrhofer et al., 2008). Hence, the positive correlation observed of the number of isolated strains of a species to the number of resistant (or multiresistant) strains and to the variety of phenotypes.

The range of the MIC values of penicillin G (<0.03–4 mg/L) in our study is similar to that reported by other researchers (D'Aimmo et al., 2007; Klare et al., 2007) but the maximum values in our study are higher than the ones reported 0.05 mg/L as the highest MIC value (Katla et al., 2001; Hummel et al., 2007). The MIC values range of ampicillin varied from 0.03 to 4 mg/L and is exactly the same as the one reported by Klare et al. (2007). Higher maximum values are reported by Salminen et al. (2006), but Danielsen and Wind (2003); D'Aimmo et al. (2007) and Katla et al. (2001) report lower maximum MIC values. The combination of ampicillin with sulbactam showed MIC values range from 0.03 to 2 mg/L, identical to the one reported by Klare et al. (2007). Comparing the resistance within the b-lactam group, 198 strains were found resistant to penicillin G distributed in eight species, 62 strains were found resistant to ampicillin distributed in three species while 91 strains were found resistant to the combination of ampicillin and sulbactam distributed in four species. It is almost certain that cross resistance phenomena occurred but not among ampicillin and ampicillin/sulbactam because the resistance to them was observed in different species. It is a rather surprising finding that the resistance against ampicillin/sulbactam was found higher than the one against ampicillin because in the Greek market medicines for veterinary use containing sulbactam do not exist. This finding is a very strong indication that the resistance against sulbactam is due to environmental contamination of the cheese with bacteria producing b-lactamase resistant to sulbactam.

Gentamycin showed a MIC range from 0.06 to 16 mg/L. Other studies report highest MIC values from 36 to 1000 mg/L (Charteris et al., 2001; Katla et al., 2001; Danielsen and Wind, 2003; D'Aimmo et al., 2007; Stsepetova et al., 2008). Researchers like Zhou et al. (2005) claim that lactobacilli do have a natural resistance against gentamicin but our results are not supporting this theory. Streptomycin showed a MIC range from 0.25 to 128 mg/L. Katla et al. (2001) report MIC range from 2 to 256 mg/L, Klare et al. (2007) from 2 to 128 mg/L (Klare et al., 2007) and Danielsen and Wind (2003) from 4 to 256 mg/L (Danielsen and Wind, 2003). Hummel et al. (2007) attribute the high MIC values to the absence from the bacterial cell of a cytochrome as an aid for electron transfer. These researchers consider lactobacilli as naturally resistant against streptomycin but again—as in the case of gentamycin- our results do not comply with their opinion. Comparing the resistance against the two aminoglycosides, it seems that the resistance against streptomycin is higher than the one against gentamycin -due to the much higher MIC values of the former- although the number of resistant strains is similar (74 and 72 respectively) distributed in exactly three species for both compounds. Cross resistance should be ruled out because resistance occurred in different species.

Several researchers consider the resistance of certain *Lactobacillus* species against Vancomycin as natural and attribute this resistance to chemical difference of peptidoglycan (Zhou et al., 2005; Ammor et al., 2008). According to Coppola et al. (2005) this finding could be used as a cheap and rapid taxonomical method of identification. Most researchers agree—and our results support this opinion- that the heterozymotic species like eg *L. pentosus* and *L. fermentum* are the resistant ones (Charteris et al., 2001; Herreros et al., 2005; Kastner et al., 2006; Salminen et al., 2006; Florez et al., 2008). In the present study the 6 homozymotic species showed a MIC range 0.12–2 mg/L while the 4 heterozymotic species showed a MIC range 0.5–128 mg/L. The value of 4 mg/L was separating the sensitive from the resistant species. Regardless of the natural resistance against Vancomycin which is usually located in the chromosome, acquired resistance—usually hosted in plasmids—has also been observed in the lactobacilli (146 strains distributed in five species). Natural resistance has also been reported for teicoplanin, another antibiotic from the glycopeptide group (Coppola et al., 2005). However, the susceptibility spectrum of teicoplanin is broader than the one of Vancomycin (Herreros et al., 2005; Mathor and Singh, 2005; Klare et al., 2007). Our results support this opinion since the value discriminating the susceptible from the resistant species is 1 mg/L. Acquired resistance concerned 84 strains distributed in two species. Cross resistance is possible among the two glycopeptides but only in the case of *L. paraplantarum* where 38 strains showed resistance to both compounds. However, 4 strains of *L. paraplantarum* and 42 strains of *L. plantarum* showed resistance against teicoplanin, indicating a plasmid located determinant. If so, then this determinant originated from the environment, given that glycopeptides are not in use in veterinary clinical practice.

Quinupristin/dalfopristin (Q/D) is a composite antibiotic representing the group of streptogramins. The lactobacilli showed a MIC range 0.03–8 mg/L. In total 139 strains belonging to six species were found resistant. Our results are similar to those of Katla et al. (2001) and Klare et al. (2007). Resistance to Q/D is achieved by neutralizing its components, a process controlled by genetic determinants in plasmids. The same determinants express resistance against the macrolides and against the lincosamides, implying that the resistance against Q/D should either be the result of cross resistance or that should be due to horizontal transfer of genes from other bacteria. Cross resistance should be ruled out because only 12 strains of *L. plantarum* show resistance against Q/D and a macrolide (clindamycin). All other strains resistant to Q/D are resistant neither to a macrolide nor to a lincosamide leading to the conclusion that the observed resistance originates from the environment.

With the exception of 42 strains of *L. paraplantarum*, all other lactobacilli were susceptible to erythromycin. The MIC range was 0.03–0.5 mg/L and in accordance with the findings of other researchers (Florez et al., 2005, 2008; Mathor and Singh, 2005; Hummel et al., 2007). Resistance to this macrolide antibiotic is controlled by genes which also control the resistance against lincosamides so it is possible that cross resistance occurred.

The Lincosamide group was represented by clindamycin. Resistance to this antibiotic was observed in 84 strains distributed in four species. The breakpoints were relatively low (0.12 mg/L for *L. salivarius* and 0.06 mg/L for the others) while the MIC range varied from 0.03 to 0.5 mg/L. These findings imply high susceptibility and agree to the ones reported by Florez et al. (2008), Klare et al. (2007), and Salminen et al. (2006). Cross resistance with Q/D and erythromycin (all acting on the 50S ribosomal subunit) cannot be ruled out in the cases of *L. plantarum* and *L. paraplantarum* but can be ruled out in the cases of *L. rhamnosus* and *L. salivarius* because the latter lactobacilli were not resistant to these antibiotics.

The tetracyclines are widely used in veterinary medicine and particularly oxytetracycline. The MIC range in the present study was from 0.06 to 32 mg/L and 60 strains distributed in five species were found resistant. Similar MIC range report and other researchers but their results on the resistance differ from ours. For example, Florez et al. (2008) report that heterozymotic lactobacilli are more resistant than the homozymotic ones, a finding that is not in agreement with our results. On the other hand Zhou et al. (2005), Coppola et al. (2005), and Charteris et al. (2001) did not observe resistance in the strains that they studied. Resistance against tetracyclines is attributed to the genetic determinants *(tet)*. There is a controversy about the incidence of these determinants as well as the exact gene but most researchers agree that the resistance to tetracyclines is acquired via plasmids (Kastner et al., 2006) or via transposons and this theory interprets our findings (Ammor et al., 2008). Cross resistance to glycosamides (the aminoglycosides and the tetracyclines act on the 30S ribosomal subunit) is possible in the cases of *L. delbrueckii* subsp *lactis* (gentamycin) and *L. fermentum* (streptomycin). The other resistant to oxytetracycline species did not include strains resistant to the aminoglycosides.

Against chloramphenicol 214 strains showed resistance, distributed in eight species. MIC ranged from 0.12 to 16 mg/L. Similar results were reported by Stsepetova et al. (2008) and Ammor et al. (2008). Resistance against chloramphenicol is controlled by the (cat) genes which are located in plasmids therefor horizontal transfer from the environment is possible (Mathor and Singh, 2005; Hummel et al., 2007). Cross resistance with other antibiotics acting on the 50S ribosomal subunit is possible in the cases of *L. paraplantarum* and *L rhamnosus* (macrolides) and in the cases of *L. helveticus, L. delbrueckii* subsp *bulgaricus*, and *L. pentosus* (Q/D).

The resistance of lactobacilli against trimethoprim is considered as natural by most researchers. Katla et al. (2001) attribute this resistance to the lack of the metabolic path of folic acid synthesis from these bacteria while Danielsen et al. (2004) regard a resistant to trimethoprim di-hydro-folate-reductase (DHFR) as responsible for the innate resistance of lactobacilli (Katla et al., 2001; Danielsen et al., 2004). However, there are some researchers who support a genetic determinant of resistance and particularly the gene (dhfr) which can be found in the chromosome as well as in a plasmid (Mascaretti, 2003). The very wide MIC range (0.5–256 mg/L) is a finding that complies with the theory of natural resistance. Furthermore 84 strains, belonging to three species were found resistant while their breakpoints varied from 1 to 4 mg/L.

The resistance of lactobacilli against metronidazole seems to be innate for this genus and all researchers agree to that (Charteris et al., 2001; Danielsen and Wind, 2003; Florez et al., 2005; D'Aimmo et al., 2007). The MIC range varied widely from 32 to >500 mg/L and the breakpoints of the 233 resistant strains (distributed in 11 species) were either 256 or >500 mg/L. These findings are in agreement with the innate resistance theory.

Fucidic acid is an inhibitor of protein synthesis and the resistance to this compound is considered as innate for lactobacilli. In *S. aureus* certain genes which control the membrane permeability to Fucidic acid are located in plasmids while another gene (fusA) is located in the chromosome (O'Brien et al., 2002). In our study the MIC range was found from 0.06 to 256 mg/L, while 66 strains (four species) were found in resistant subpopulations. The very wide MIC range is a finding compatible to the innate resistance theory.

A thorough review of our results reveals that the observed resistance can be attributed to cross resistance phenomena in many cases. This is expected for antibiotics with similar chemical structure like e.g. the b-lactam group (penicillin G and ampicillin) or with the same mode of action in the bacterial cell like e.g., the lincosamide group and the macrolide group acting on the 50S ribosomal subunit (Table S3 shows the most probable cross resistance patterns). Yet, a great part of our results cannot be attributed to cross resistance as we have already discussed.

Foods link—in many levels—the consumers and the environment in which they are produced. Resistant bacteria isolated from various foods reflect the microbial resistant of their ecosystem. Thus, foods can be considered as indicators of the environmental resistance to antibiotics. Cheeses can be regarded as complex and dynamic ecosystems of microbial populations and are subsystems of their production environment reflecting its impacts. Resistant bacteria can transfer their resistance determinants from nature to the consumers via the food chain (EFSA, 2008; Lord Soulsby of Swaffham Prior, 2008; Mayrhofer et al., 2008; Silveira et al., 2009; Leclercq et al., 2011). This can be achieved by spoilage contamination through the dairy process when not pathogenic microorganisms transfer this resistance horizontally to the cheese flora. The entrance of new animals in the house hold, the exchange of equipment and tools, the wind and the dust, the insects, the rodents, the wild birds, and of course the humans are the major carriers of resistant microorganisms.

Mykonos though a small island is one of the most recognized tourist destinations worldwide. Hundreds of thousands of tourists swarm the island every year and assist involuntarily in the circulation and the dissemination of such genes. Resistant against penicillin G, oxytetracycline, and aminoglycosides is an expected finding because of their extensive use (and misuse) to the livestock but the resistance against macrolides and against lincosamides (let alone the resistance against streptogramins and glycopeptides) is an alarming finding because these antibiotics are either rarely used in veterinary clinical practice or not at all. The latter groups of antibiotics are opwever, extensively used in the community or in hospitals.

Traditional artisan foods—like the Kopanisti cheese—are produced in households and every household represents an ecological singularity creating numerous niches which lead to the impressive biodiversity of the isolated *Lactobacillus* species (Amador et al., 2009). Yet, the same ecological parameters permit the circulation of other microorganisms too, of environmental origin, bearing resistance genes. The horizontal transfer of resistance between two bacteria is donor and receiver specific (Xinhui et al., 2011). Wide biodiversity implies that there are increased probabilities for the donors and the receivers to interact. This transfer can potentially take place in the intestinal tract of the consumer. Gevers et al. (2003) isolated lactobacilli resistant to tetracyclines from sausage and studied the *in vitro* horizontal transfer of this resistance to *Enterococcus feacalis* and to *Lactococcuslactis* (Gevers et al., 2003). Phillips et al. (2004) argue that more epidemiological studies are required to understand the mechanisms of foodborne resistance (Phillips et al., 2004). The same researchers also argue that bacteria bearing resistance to antibiotics need only a short time to survive in the consumer's gut in order to transmit the resistant elements. Hence, potential public health issues arise from the consumption of foods like Kopanisti cheese. The establishment of resistance to the intestinal flora poses the threat to transfer this resistance to future pathogens, yet this threat remains potential and stochastic (Wickens and Wade, 2005; IFT Expert Report, 2006).

## Conclusion

The aim of this study was to assess the resistance of lactobacilli isolated from the traditional artisan cheese Kopanisti. Our results clearly show that resistance was observed against all testes antibiotics. Furthermore, the isolated multiresistant strains were in alarmingly high levels (63.76% of the total). Given that the cheese is artisan and produced in rural households and that part of the phenotypes expressed were against antibiotics that are not used in the veterinary clinical practice, our findings are strong evidences that this resistance is at large due to environmental contamination during the ripening of the cheese. The possibility of transmission of this resistance to the consumers of the cheese, though possible, must be assessed by further epidemiological studies.

## Author contributions

GR, CV, and ES performed research. CV, AT, AA, and IS analyzed data and contributed to discussion. EB conceived the study.

### Conflict of interest statement

The authors declare that the research was conducted in the absence of any commercial or financial relationships that could be construed as a potential conflict of interest.
