# Supplementary material for: Biodiversity and Microbial Resistance of Lactobacilli Isolated From the Traditional Greek Cheese Kopanisti
Source: Front Microbiol. 2018 Mar 22;9:517. doi: 10.3389/fmicb.2018.00517 (PMC5875084; doi:10.3389/fmicb.2018.00517)
Supplement: Supplementary file 1 [file Table1.PDF]

Species/antibiotic	**Pen G**	**Amp**	**Amp/Sul**	**Ery**	**Clin**	**Clor**	**Gen**	**Stre**	**Van**	**Tei**	**Clor**	**Gen**	**Stre**	**Van**	**Tei**
***L. helveticus* (n=31)**	-	-	-	-	-	27	-	-	-	-	27	-	-	-	-
***L. acidophilus* (n=71)**	65	-	66	-	-	12	-	-	36	-	12	-	-	36	-
***L. paraplantarum* (n=48)**	-	-	-	42	36	30	-	30	38	42	30	-	30	38	42
***L. brevis* (n=30)**	-	-	5	-	-	-	-	-	-	-	-	-	-	-	-
***L. delbrueckii susbsp bulgaricus* (n=84)**	31	36	-	-	-	66	42	-	54	-	66	42	-	54	-
***L. johnsonii* (n=49)**	20	-	-	-	-	30	-	25	-	-	30	-	25	-	-
***L. curvatus* (n=42)**	31	19	-	-	-	-		-	12	-	-		-	12	-
***L. salivarius* (n=12)**	6	-	6	-	5	-	-	-	-	-	-	-	-	-	-
***L. plantarum* (n=54)**	33	-		-	12	-	-	-	-	42	-	-	-	-	42
***L. rhamnosus* (n=30)**	-	-	14	-	13	24	24	-	6	-	24	24	-	6	-
***L. . delbrueckii susbsp lactis* (n=36)**	6	-	-	-	-	-	6	-	-	-	-	6	-	-	-
***L. fermentum* (n=25)**	-	-	-	-	-	-	-	19	-	-	-	-	19	-	-
***L. pentosus* (n=13)**	-	-	-	-	-	7	-	-	-	-	7	-	-	-	-
***L. casei subsp casei* (n=12)**	-	-	-	-	-	-	-	-	-	-	-	-	-	-	-
***L. reuteri* (n=7)**	-	-	-	-	-	-	-	-	-	-	-	-	-	-	-
***L. casei subsp pseudoplantarum* (n=6)**	-	-	-	-	-	-	-	-	-	-	-	-	-	-	-
***L. sakei*(n=24)**	6	7	-	-	-	18	-	-	-	-	18	-	-	-	-
**Total (n=574)**	198	62	91	42	84	214	72	74	146	84	214	72	74	146	84

Table 1: Isolated *Lactobacillus* species and resistant strains from Kopanisti cheese

Clor: Chloramphenicol, **Gen**: Gentamycin, **Stre**: Streptomycin, **Van:** Vancomycin, **Tei:** Teicoplanin. **Met:** Metronidazole, **Oxy**: Oxytetracycline, **Tri:** Trimethoprim, **Fus**: Fucidic acid, **Q/D**: Quinupristin/Dalfopristin, **Pen G**: Penicillin G, **Amp**: Ampicillin, **Amp/Sul**: Ampicillin/Sulbactam, **Ery**: Erythromycin, **Clin**: Clindamycin
