# Supplementary material for: Biodiversity and Microbial Resistance of Lactobacilli Isolated From the Traditional Greek Cheese Kopanisti
Source: Front Microbiol. 2018 Mar 22;9:517. doi: 10.3389/fmicb.2018.00517 (PMC5875084; doi:10.3389/fmicb.2018.00517)
Supplement: Supplementary file 2 [file Table2.PDF]

Species	Sensitive	Resistant *	Multiresistant **	Total (n=574)	Antibiotics***
L. helveticus	4	20	7	31	4
L. acidophilus	5	1	65	71	6
L. paraplantarum	5	-	43	48	8
L. brevis	19	11	-	30	2
L. delbrueckii susbsp bulgaricus	4	13	67	84	7
L. johnsonii	23	2	24	49	4
L. curvatus	9	3	30	42	6
L. salivarius	-	-	12	12	5
L. plantarum	7	5	42	54	6
L. rhamnosus	6	-	24	30	6
L. . delbrueckii susbsp lactis	18	4	14	36	4
L. fermentum	6	6	13	25	3
L. pentosus	6	-	7	13	3
L. casei subsp casei	6	6	-	12	1
L. reuteri	-	-	-	7	-
L. casei subsp pseudoplantarum	-	-	-	6	-
L. sakei	5	1	18	24	5
**Total**	123 (21.42%)	72 (12.54%)	366 (63.76%)		

Table 2: Sensitive, resistant and multiresistant *Lactobacillus* strains isolated from Kopanisti cheese

- *Resistant to one antibiotic
- ** Resistant to at least two antibiotics
- ***Number of antibiotics against which resistance was observed within the strains of a species
