# Supplementary material for: Biodiversity and Microbial Resistance of Lactobacilli Isolated From the Traditional Greek Cheese Kopanisti
Source: Front Microbiol. 2018 Mar 22;9:517. doi: 10.3389/fmicb.2018.00517 (PMC5875084; doi:10.3389/fmicb.2018.00517)
Supplement: Supplementary file 3 [file Table3.PDF]

Antibiotic	Mode of action	Resistant species (Resistant strains) a
Penicillin G	Inhibition of bacterial cell wall synthesis	8 (198)
Ampicillin		3 (62)
Ampicillin/Sulbactam		4 (91)
Chloramphenicol	Inhibition of protein synthesis through connection with the 50S ribosomal subunit	8 (214)
Clindamycin		4 (84)
Vancomycin		5 (146)
Teicoplanin		2 (84)
Erythromycin		1 (42)
Quinupristin/ Dalfopristin		6 (139)
Gentamycin	Inhibition of protein synthesis through connection with the 30S ribosomal subunit	3 (72)
Streptomycin		3 (74)
oxytetracycline		5 (60)
Trimethoprim	Inhibition of the metabolic path of the folic acid	3 (84)
Metronidazole	DNA destruction	11 (233)
Fucidic acid	Inhibition of protein synthesis	4 (66)

Table 3: Antibiotics used in the present study, their modes of action, resistant species and resistant strains
