# Supplementary material for: Biodiversity and Microbial Resistance of Lactobacilli Isolated From the Traditional Greek Cheese Kopanisti
Source: Front Microbiol. 2018 Mar 22;9:517. doi: 10.3389/fmicb.2018.00517 (PMC5875084; doi:10.3389/fmicb.2018.00517)
Supplement: Supplementary file 4 [file Table4.PDF]

Species/antibiotic	**Pen G**	**Amp**	**Amp/Sul**	**Er**	**Clin**	**Clor**	**Gen**	**Stre**	**Van**	**Tei**	**Met**	**Oxy**	**Tri**	**Fus**	**Q/D**
***L. helveticus* (n=31)**	-	-	-	-	-	0.25	-	-	-	-	>500	1	-	-	0.12
***L. acidophilus* (n=71)**	0.12	-	0.12	-	-	2	-	-	0.5	-	>500	-	4	-	-
***L. paraplantarum* (n=48)**	-	-	-	0.12	0.06	1	-	2	16	8	>500	-	-	4	-
***L. brevis* (n=30)**	-	-	0.5	-	-	-	-	-	-	-	-	2	-	-	-
***L. delbrueckii* susbsp *bulgaricus* (n=84)**	0.5	0.5	-	-	-	4	2	-	0.25	-	>500	-	-	-	0.06
***L. johnsonii* (n=49)**	0.25	-	-	-	-	2	-	2	-	-	>500	-	-	-	-
***L. curvatus* (n=42)**	0.25	0.5	-	-	-	-		-	32	-	>500	0.5	-	-	1
***L. salivarius* (n=12)**	0.12	-	0.12	-	0.12	-	-	-	-	-	256	-	-	0.25	-
***L. plantarum* (n=54)**	0.5	-		-	0.06	-	-	-	-	16	>500	-	-	4	0.25
***L. rhamnosus* (n=30)**	-	-	0.25	-	0.06	1	0.5	-	>500	-	256	-		-	-
***L.delbrueckii* susbsp *lactis* (n=36)**	0.12	-	-	-	-	-	4	-	-	-	256	0.5	-	-	-
***L. fermentum* (n=25)**	-	-	-	-	-	-	-	2	-	-	-	1	-	-	0.25
***L. pentosus* (n=13)**	-	-	-	-	-	2	-	-	-	-	-	-	2	-	0.12
***L. casei* subsp *casei* (n=12)**	-	-	-	-	-	-	-	-	-	-	256	-	-	-	-
***L. reuteri* (n=7)**	-	-	-	-	-	-	-	-	-	-	-	-	-	-	-
***L. casei subsp pseudoplantarum* (n=6)**	-	-	-	-	-	-	-	-	-	-	-	-	-	-	-
***L. sakei*(n=24)**	0.12	0.12	-	-	-	0.25	-	-	-	-	-	-	1	0.25	-

Table 4: The breakpoints of the *Lactobacillus* species isolated from Kopanisti cheese (in mg/L)

Clor: Chloramphenicol, **Gen**: Gentamycin, **Stre**: Streptomycin, **Van:** Vancomycin, **Tei:** Teicoplanin. **Met:** Metronidazole, **Oxy**: Oxytetracycline, **Tri:** Trimethoprim, **Fus**: Fucidic acid, **Q/D**: Quinupristin/Dalfopristin, **Pen G**: Penicillin G, **Amp**: Ampicillin, **Amp/Sul**: Ampicillin/Sulbactam, **Er**: Erythromycin, **Clin**: Clindamycin
